# Effect of Wholewheat Flour Particle Shape Obtained by Different Milling Processes on Physicochemical Characteristics and Quality of Bread

**DOI:** 10.17113/ftb.58.03.20.6766

**Published:** 2020-09

**Authors:** José Luis Navarro, Malena Moiraghi, Fernanda Micaela Quiroga, Alberto Edel León, María Eugenia Steffolani

**Affiliations:** 1 Institute of Food Science and Technology of Córdoba (ICYTAC), CONICET-UNC Valparaiso and Rogelio Martínez Avenue, 5000 Córdoba, Argentina; 2 Department of Biological Chemistry, Faculty of Agricultural Sciences, National University of Córdoba (UNC), Valparaiso and Rogelio Martínez Avenue, 5000 Cordoba, Argentina

**Keywords:** wholewheat flour, wholegrain milling, particle size and shape, thermogravimetric analysis, bread volume

## Abstract

**Research background:**

Wholewheat flour is a very good source of nutritional compounds and functional ingredients for human diet. However, it causes negative effect on bread quality. Different milling techniques can be used to obtain wholewheat flour, minimizing the negative effect of both bran and germ on bread quality. The aim of this work is to study the effect of particle size and shape of wholegrain flour on the interaction among the different components, water distribution, dough rheology and bread volume.

**Experimental approach:**

Wholewheat flour of three varieties (Klein Rayo, Fuste and INTA 815) was obtained in cyclonic, hammer and roller mills. The characteristics of wholewheat flour were explored, and the water distribution and rheological properties of dough were determined by thermogravimetric analysis and Mixolab test, respectively. Finally, microscale bread was prepared.

**Results and conclusions:**

The amount of water-soluble pentosans, damaged starch and wet gluten was affected by the milling procedure. Regarding dough rheological properties, wholewheat flour obtained in hammer mill had the lowest water absorption capacity and the highest developing time. This result could be mainly attributed to particle shape in these samples with large amount of endosperm attached to the bran, hindering protein unfolding. Thermogravimetric analysis showed that both fine and large bran particle size seem to have the same effect on water properties in wholewheat dough during heating. Bread made with Klein Rayo variety had the highest specific volume, indicating that wheat with high protein content and breadmaking quality is needed to make wholewheat bread. The results of this work showed that particle shape, rather than particle size, affected the quality of wholewheat flour for breadmaking.

**Novelty and scientific contribution:**

The effect of milling type and particle shape of the wholewheat flour had a greater effect than the wheat variety. Thus, the wholegrain milling process should be carefully selected taking to account the shape of the produced particle. This may open new opportunities for developing wholewheat bread with better acceptance by consumers.

## INTRODUCTION

At present, consumers are trying to change their dietary habits in an attempt to gain health benefits and prevent future diseases. In this sense, consumers are open to exploring healthy alternatives that were mostly rejected in the past. Wholegrains are a nutritional option with increasing acceptance, and this is recognised by the food industry. Moreover, whole grain consumption is recommended by the World Health Organization for a healthy diet with the aim of preventing a range of non-communicable diseases (*1*).

Wholewheat flour (WWF) is a very good source of nutritional compounds and functional ingredients for human diet. Unlike refined flour, WWF is rich in ﬁbre, antioxidants, vitamins, minerals and other phytochemicals such as carotenoids, ﬂavonoids and phenolic acids (*2*). In addition, the intake of wholegrains is associated with a decreased risk of cardiovascular diseases, diabetes, obesity and colon cancer (*3*, *4*).

Many research works have proved that bread made with wholewheat flour had reduced technological quality as compared to that made with refined flour (*5*, *6*). The main reasons for this detrimental effect on bread quality have been attributed to: (*i*) water-holding capacity of bran limiting gluten network development, (*ii*) gluten dilution effect and (*iii*) disruption of gas cells (*7*). Moreover, Every *et al.* (*8*) confirmed that the germ contains reducing compounds such as glutathione, which depolymerizes gluten network. Pareyt *et al.* (*9*) also found high levels of non-polar lipids, which tend to destabilize gas cells and thus decrease loaf volume.

One strategy that has been explored is the reduction in bran particle size, thus decreasing its steric hindrance during gluten development. However, the obtained results are controversial and inconclusive. While some studies found that the reduction of bran particle size improved bread volume (*10*), Noort *et al*. (*5*) reported a negative effect when bran particle size was smaller than or equal to that of starch granules, arguing that fibre negatively affects the formation of gluten network by a combination of both physical and chemical mechanisms. On the other hand, Coda *et al*. (*11*) and Bressiani *et al.* (*4*) reported that there is an optimal particle size for whole flour, and that this allowed producing bread of acceptable quality.

The characteristics of wholewheat flour can be largely influenced by the milling process. Different milling techniques can be used to produce wholewheat flour, minimizing the negative effect of both bran and germ on bread quality. One type of milling is hammer milling, where wheat grains are impacted between a wall and a hammer to reduce its particle size according to the sieve selected by users (*12*). Another milling procedure is the cyclonic mill. The wheat sample is ground at high speed by impacting the kernels against an abrasive surface. The cyclone cools the wholewheat flour so that its properties are not modified. Next option is the roller mill, which can be used to produce flour while separating bran and germ, and then recombined to obtain wholewheat flour with the same relative proportion as in intact grains.

Liu *et al.* (*13*) studied the effect of different milling treatments of wholewheat flour on the quality of steamed bread; however, they obtained the initial flour by using a roller mill and then subjecting germ and brain to different milling types (hammer, stone, ultrafine and recombining processes). Although many studies have already analysed the effect of flour particle size on bread technological properties, few studies have examined the effect of the particle shape of wholewheat flour.

Due to this lack of agreement, the objective of this work is to study the effect of particle size and shape of wholegrain flour on the interaction among the different components, dough rheology and bread volume. In this sense, wholewheat flour (WWF) was obtained by different milling processes, and its shape and size were analysed. In addition, the characteristics of WWF were explored and the water distribution and rheological properties of dough were determined by thermogravimetric analysis and Mixolab test, respectively.

## MATERIALS AND METHODS

### Wholewheat flour

Three varieties of wheat samples, Klein Rayo, Fuste and INTA 815, were provided by Instituto Nacional de Tecnología Agropecuaria (INTA, Marcos Juárez, Argentina) and harvested in 2016. The varieties used in this work are classified according to the genetic quality established by the Winter Cereal Committee of the National Seed Commission (Argentina) (*14*) in three groups with annual update. The Klein Rayo variety (composition in % on dry mass basis: proteins 12.65, ash 2.05, lipids 3.41) is a wheat used as a corrector with very strong gluten, while Fuste variety (composition in % on dry mass basis: proteins 10.81, ash 1.85, lipids 3.74) is a good-quality wheat used for long fermentation breadmaking. INTA 815 (composition in % on dry mass basis: proteins 11.42, ash 1.90, lipids 3.03) has high flour yield but low breadmaking quality. Grains were ground to obtain wholewheat flour (WWF) with three different mills, namely cyclonic mill (Cyclotec^TM^ 1093; Foss, Hillerød, Denmark) using a 1-mm mesh sieve, hammer mill (Pulverisette 16; Fritsch, Idar-Oberstein, Germany) with a 1-mm mesh sieve, and roller laboratory mill (Mill CD1; Chopin Technologie, Villeneuve-la-Garenne, France). With this last type of mill, all the millstreams (bran, germ and endosperm) were recovered and recombined together to obtain wholewheat flour. Thus, all flour samples had the same proportion of bran, germ and endosperm as the original wheat grain. The chemicals used were of analytical grade. The ingredients employed in the preparation of the microscale bread were purchased in the local market.

### Particle size determination

Particle size distribution of WWF was determined by laser light diffraction (Horiba LA 960, Kyoto, Japan) in triplicate in 0.2 g of sample in aqueous suspension. The *d*_10_, *d*_50_ and *d*_90_ corresponding to the maximum diameter of 10, 50 and 90% of the particles in the total volume, respectively, were calculated. In addition, average particle size and span were calculated, providing information on the amplitude and heterogeneity of the distribution:

Span=(*d*_90_-*d*_10_)/*d*_50_ /1/

### Scanning electron microscopy

The microstructure of wheat flour particles was studied using scanning electron microscopy (SEM). Samples were dehydrated with phosphate buffer (0.1 mol/L, pH=6.8), ethanol (30, 50, 70, 80 and 90%) and subjected to vacuum. The samples were sprinkled onto double-sided tape attached to the specimen stubs and coated with a thin layer of gold (30 nm thickness) through a cathodic spray coating system. For the observations, an electronic scanning microscope (FE-SEM Σigma, Carl Zeiss, Oberkochen, Germany) was used under high vacuum conditions (10^-4^ Pa) at an acceleration voltage of 3.00 kV. Images were obtained at 333× magnification.

### Bran images by stereomicroscopy

Bran particles were resuspended and washed with distilled water. They were then dried in a stove for 4 h at 40 °C and observed with a stereo microscope S8AP0 (Leica Microsystems Inc., Bannockburn, IL, USA). The resulting images were analysed using ImageJ v. 1.51j8 software (*15*) to calculate and display shape descriptors, such as area, perimeter and circularity.

### Characteristics of wholewheat flour

Moisture, ash and lipid content of the samples were measured according to the approved AACC methods 44-15.02 (*16*), 08-12.01 (*17*) and 30-25.01 (*18*), respectively. Briefly, moisture content was determined by weighing the sample prior to and after drying for 2 h at 130 °C (dry oven model 600 D060602; Memmert, Schwabach, Germany). Ash content was determined by weighing the sample prior to and after igniting for 2 h at 600 °C (model 332; Indef, Córdoba, Argentina). Determination of total lipid was done by Soxhlet extraction with petroleum ether (Sintorgan, Buenos Aires, Argentina). After the extraction, lipid content was determined by weighing. Protein content (Kjeldahl method 46-12.01 (N×5.7) (*19*)) was determined after their digestion with concentrated H_2_SO_4_ (Sintorgan, Buenos Aires, Argentina). Digestion was performed in Raypa digestor (Barcelona, Spain) and distillation in UDK 126A (VELP, Scientifica, Milan, Italy). The damaged starch content was evaluated according to AACC 76-31.01 method (*20*). Wet gluten content was obtained according to the hand washing method 38-10.01 (*21*). The content of total and water-soluble pentosans in flour were quantified following the orcinol-HCl method described by Steffolani *et al.* (*22*) at 670 nm with UV-Vis spectrometer (model V-730; JASCO Mary's Court Easton, MD, USA). Wholewheat flour was analysed using a prediction test developed for refined flour. The hydration capacity of the proteins in an acidic environment was determined by means of the sodium dodecyl sulphate (SDS) sedimentation index according to Moiraghi *et al*. (*23*).

### Evaluation of mixing and pasting properties by Mixolab

The mixing and pasting behaviour tests of WWF were carried out under controlled heating conditions in a Mixolab analyser (Tripette et Renaud, Chopin Technologies, Villeneuve-la-Garenne, France) according to the method 54-60.01 (*24*). A certain amount of water was added to each sample depending on water absorption capacity to reach the maximal 1.1 N∙m, representing 500 Brabender units of dough consistency. The parameters obtained from the Mixolab included water absorption capacity and dough properties, such as dough development time (C1), protein weakening (C2), starch gelatinization (C3), stability of hot starch paste (C4) and starch gelling (C5).

### Breadmaking procedure

According to Moiraghi *et al.* (*23*), microscale bread tests were carried out with 20 g flour with minor modifications for wholewheat flour. The ingredients used were (on 100 g flour basis): NaCl 2%, sucrose 1%, dry baker’s yeast 1% and optimum water level (water absorption by Mixolab). Ingredients were mixed for 2 min in a manual mixer (Supermix 130; Moulinex, Buenos Aires, Argentina). The resulting dough was taken to a first proof for 20 min at 30 °C in a water-saturated atmosphere. The dough was then manually degassed and sheeted with a Pastalinda® machine (Buenos Aires, Argentina) to form an oval dough piece. This was folded twice in half. The dough was then divided into 10-g pieces, rolled up and placed in a baking pan (40 mm×25 mm×20 mm). After fermentation for 35 min at 30 °C in a water-saturated atmosphere, dough was baked for 12 min at 200 °C. The volume of each bread loaf was determined by the rapeseed displacement method (2 h after baking (*25*). Specific bread volume was calculated as the ratio of bread volume to bread mass.

### Thermogravimetric analysis

The thermal properties of wholewheat dough from Klein Rayo flour obtained by different milling methods were analysed by thermogravimetric analysis (TGA) in a Discovery TGA (TA Instruments, New Castle, DE, USA). Dough samples were prepared according to bread formulation (without sugar and yeast) and breadmaking procedure. Dough samples (~35 mg) were heated in aluminium pans under a nitrogen atmosphere (nitrogen flow rate 50 mL/min) at a heating rate of 5 °C/min from 25 to 150 °C. Each run was repeated at least twice. All the thermogravimetric traces, namely mass loss *vs* temperature, were calculated based on the initial water content of each dough sample. From these thermogravimetric traces, we determined temperature at which samples lost 75, 80 and 90% water and percentage of total water loss at several temperatures. The first derivative of the thermogravimetric traces was then determined, representing the rate of water loss (derivative thermogravimetry (DTG, %/°C) using TRIOS v. 4.3.1 (*26*) to identify specific water loss events. In addition, DTG traces were fitted to a sum of Gaussian functions using PeakFit v. 4.12 (*27*) with the aim of determining different water types (*i.e.* free and bound to each major component). The Gaussian peaks were initially added around peak centres and the final location and Gaussian area were determined by automatic fitting to get the best fit to the data. Peak area was expressed as a percentage of the total area under the curve. Adjustments with regression coefficient (r^2^) greater than 0.99 were considered.

### Statistical analysis

Results were obtained at least in duplicate and expressed as the mean value±standard deviation. The data obtained for the same wheat variety were evaluated by analysis of variance (ANOVA) and the results were compared by Di Rienzo, Guzmán and Casanoves (DGC) multiple-comparison test at a significance level of 0.05. In addition, a variance analysis was performed considering the mean value of each treatment (milling process). All analyses were performed using the INFOSTAT statistical software (*28*).

## RESULTS AND DISCUSSION

### Wholewheat flour particle shape and size

The particle size distribution of wholewheat flour was different depending on the milling procedure (Table 1). Particle size data can be slightly overestimated due to the hydration of particles. However, all the samples were subjected to the same conditions for measurement. The wholewheat flour (WWF) obtained with the cyclonic mill was characterized by a relatively small particle size distribution (*d*_90_ between 519 and 648 µm) with a span of 3.77-4.44. The *d*_90_ of the WWF obtained in the hammer mill showed values between 1079 and 2344 µm with a span of 1.84-2.36. The roller mill allowed obtaining WWF with large particle size (1534-4167 µm); however, span was grain variety-dependent, where Klein Rayo had the highest span, and INTA 815 the lowest. In the first step of roller milling process, wheat grains were crushed through serrated rollers that tore and triturated the grain. In the second step, endosperm particles were reduced in size. The bran particles obtained by this milling process had a large size since the inclined rollers completely separated the bran histological layers from the endosperm. The resulting flour had an endosperm reduced in size, but with greater germ and bran particles.

**Table 1:** Particle size distribution of wholewheat flour and bran shape descriptors

Variety	Mill type	Particle size distribution		Bran particle shape			
*d*_90_/µm	Span	*A*/mm^2^	*P*/mm	Circularity
Klein Rayo	Cyclonic	(626±1)^a^	(3.8±0.2)^b^	(0.38±0.03)^a^	(3.48±0.05)^a^	(0.39±0.01)^b^
	Hammer	(1079±44)^b^	(1.87±0.06)^a^	(0.37±0.01)^a^	(3.74±0.01)^a^	(0.36±0.00)^b^
	Roller	(1534±10)^c^	(11.6±0.3)^c^	(2.4±0.3)^b^	(10.8±0.3)^b^	(0.24±0.03)^a^
Fuste	Cyclonic	(648±68)^a^	(4.3±0.5)^b^	(0.3±0.2)^a^	(3.1±0.7)^a^	(0.41±0.00)^b^
	Hammer	(2080±58)^b^	(1.84±0.07)^a^	(0.6±0.2)^a^	(4.1±0.3)^b^	(0.37±0.04)^a^
	Roller	(1944±33)^c^	(5.3±0.1)^b^	(1.7±0.3)^b^	(8.7±0.7)^b^	(0.29±0.02)^a^
INTA 815	Cyclonic	(519±13)^a^	(4.44±0.05)^c^	(0.52±0.03)^a^	(5.09±0.03)^a^	(0.27±0.02)^a^
	Hammer	(2344±48)^b^	(2.36±0.01)^b^	(0.46±0.07)^a^	(3.7±0.3)^a^	(0.42±0.03)^b^
	Roller	(4167±5)^c^	(319±0.1)^a^	(1.9±0.4)^b^	(8.5±0.7)^b^	(0.35±0.01)^b^
Mean*	Cyclonic	(582±71)^a^	(4.2±0.4)^b^	(0.4±0.1)^a^	(3.9±1.1)^a^	(0.36±0.08)^b^
	Hammer	(1117±51)^a^	(2.0±0.3)^a^	(0.46±0.09)^a^	(3.9±0.2)^a^	(0.38±0.03)^b^
	Roller	(2699±1130)^b^	(677±3.9)^b^	(2.0±0.3)^b^	(9.3±1.2)^b^	(0.29±0.05)^a^

*A*=area, *P*=perimeter. Different letters within a column for the same wheat variety indicate that values are significantly different at p<0.05. *Values of each milling treatment represent the average of the three varieties

Conversely, the cyclonic mill caused a homogenous reduction of WWF particle size in a single step. The principle of this milling process is a turbine wheel that spins at a very high speed, breaking the sample into pieces and hurling them out to the rim where they are abraded to a fine dust.

**Fig. 1:**
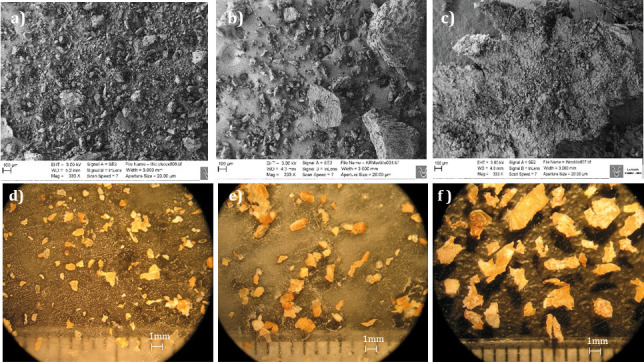
Scanning electron micrographs of: a-c) wholewheat flour and d-f) stereo microscopy images of bran particles of Klein Rayo variety obtained in: a and d) cyclonic, b and e) hammer and c and f) roller mill

The mechanism of hammer mill is an intermediate between the roller and cyclonic mills; the speed is lower than that of cyclonic mill, without abrading rim. As a consequence, the WWF particles obtained by hammer mill had medium? size, compared to particles obtained by roller and cyclonic mills, and a large amount of endosperm attached to the bran (Fig. 1).

In general, the shape of bran particles can be represented as a combination of magnitudes such as area and perimeter or by a single magnitude that indicates the percentage of similarity to a given geometric object such as circularity. This shape descriptor is a measure of a circle and ranges from 0.0 to 1.0, where 1.0 is a perfect circle (*29*).

The analysis of particle shape allowed determining the homogeneity between the WWF obtained with the cyclonic mill and the heterogeneity of the particles obtained by roller mill. In this sense, the WWF obtained by roller mill had particles with the highest perimeter and surface area, whereas circularity was the lowest. In addition, the bran particles obtained by roller mill had irregular shapes and the typical structure of histological tissues, while the bran particle obtained from hammer and cyclonic mills lost part of their original structure (*30*). As Saad *et al*. (*31*) described the milling process, an erosion phenomenon occurs, the outer surface of the irregular bran particles may undergo a friction, causing the small irregularities on the surface to disappear and generating particles with more regular shapes.

### Wholewheat flour characterization

The mass fractions of proteins, lipids and ash in the WWF were not affected by the milling type since it was obtained from the same varieties. However, the form in which some specific components appear depended on the type of milling (Table 2). Starch is damaged by mechanical action during wheat milling of starch granules. The damaged granules negatively affect dough behaviour and quality of breadmaking flour (*32*). In this work, the wholewheat flour obtained in hammer mill had the lowest damaged starch mass fraction of the three varieties, while WWF obtained in cyclonic and roller mills had similar mass fraction of damaged starch. These results confirmed that the hammer mill breaks wheat grain without tearing it; as a consequence, the endosperm adhered to bran suffers less damage.

**Table 2:** Characteristics of wholewheat flour

		*w*/%				
Variety	Mill type	DS	TP	WSP	WG	*V*(SDS-SI)/mL
Klein Rayo	Cyclonic	(7.9±0.0)^b^	(11.4±0.1)^a^	(0.70±0.07)^b^	(26.2±0.0)^b^	(10.6±0.4)^b^
	Hammer	(3.4±0.4)^a^	(11.5±0.4)^a^	(0.53±0.01)^a^	(20.9±0.8)^a^	(7.0±0.0)^a^
	Roller	(8.3±0.0)^b^	(11.3±0.5)^a^	(0.77± 0.00)^b^	(24.8±0.0)^b^	(11.8±0.4)^c^
Fuste	Cyclonic	(8.5±0.3)^b^	(9.4±0.9)^a^	(0.61±0.02)^b^	(24.6±0.3)^c^	(9.2±0.4)^b^
	Hammer	(3.4±0.6)^a^	(10.0±1.5)^a^	(0.43±0.01)^a^	(16.1±0.3)^a^	(5.8±0.4)^a^
	Roller	(8.5±0.3)^b^	(10.4±0.7)^a^	(0.57±0.01)^b^	(22.0±0.0)^b^	(11.0±0.0)^c^
INTA 815	Cyclonic	(7.4±0.0)^b^	(13.0±2.1)^a^	(0.85±0.04)^a^	(27.4±0.1)^b^	(10.1±0.2)^b^
	Hammer	(3.6±0.3)^a^	(12.7±0.6)^a^	(0.7±0.04)^a^	(20.0±0.8)^a^	(6.3±0.0)^a^
	Roller	(8.2±0.1)^b^	(12.7±1.9)^a^	(0.85±0.02)^a^	(25.2±0.2)^b^	(12.0±0.0)^c^
	Cyclonic	(7.9±0.5)^b^	(11.3±1.9)^a^	(0.7±0.12)^b^	(26.1±1.2)^b^	(10.0±0.6)^b^
Mean*	Hammer	(3.5±0.3)^a^	(11.4±1.4)^a^	(0.6±0.13)^a^	(19.0±2.3)^a^	(6.3±0.6)^a^
	Roller	(8.3±0.2)^b^	(11.5±1.4)^a^	(0.73±0.09)^b^	(24.0±1.6)^b^	(11.6±0.5)^c^

DS=damaged starch, TP=total pentosans, WSP=water-soluble pentosans, WG=wet gluten, SDS-SI=sodium dodecyl sulphate sedimentation index. Different letters within a column for the same wheat variety indicate values are significantly different at p<0.05. Results are expressed on dry mass basis. *Values of each milling treatment represent the average of the three varieties

The effect of milling on the total pentosan mass fraction of WWF was not significantly influenced by the type of milling, and this result was expected since milling was integral. However, the total pentosan mass fraction showed significant differences among varieties: INTA 815 had the highest value and Fuste the lowest. On the other hand, the soluble pentosan mass fraction depended on the milling type; the WWF from hammer mill had the lowest soluble pentosan mass fraction compared to other milling processes. The high water-extractable pentosan in WWF obtained in cyclonic and roller mills could be attributed to the rupture of the cell wall, resulting in a release of pentosan polymers entangled in the cell wall matrix. In addition, the friction on the grinding ring of cyclonic mill might result in the cleavage of covalent bonds, turning water-unextractable pentosan into water-extractable pentosan (*33*).

Wet gluten mass fraction was determined by the hand washing method since the glutomatic method did not allow developing good network and full washing. The WWF obtained in cyclonic and roller mills had significantly higher wet gluten mass fraction compared to the WWF produced in hammer mill. This result indicated a significant effect of milling type on the quality of breadmaking flour, whereas the particle size of WWF and wheat variety had a minor effect. The gluten network is formed and stabilized by covalent disulphide bonds and non-covalent interactions such as hydrogen bonds, ionic bonds and hydrophobic bonds between gliadin and glutenin (*34*), and bran and germ particles interfere during the development of this structure. The SDS sedimentation index is a predictive test of the quality of breadmaking flour. Wholewheat flour had lower sedimentation index than white flour, and this result is mainly attributed to the lower gluten mass fraction in WWF. In this work, different varieties showed no effect on SDS sedimentation test, yet the number of samples analysed was very low. The milling method probably had the biggest influence in this test. Morris *et al.* (*35*) examined the SDS sedimentation test on wholewheat flour and observed that this assay was highly sensitive to differences among hexaploid ‘bread’ wheat. In the same work, the authors found no effect between grinding type and particle size. In our work, the WWF obtained in roller mill showed the highest sedimentation index, followed by cyclonic mill, whereas the lowest sedimentation volume in WWF was obtained with the hammer mill. Therefore, the particle shape of the flour from the hammer mill, with large amount of endosperm attached to the bran, could hinder protein unfolding by SDS and the floccules formed were thus small, unstable and heavier, and their volume sedimentation was low.

### Rheological properties of dough samples

The properties of dough were analysed with a Mixolab (Table 3). This equipment allows simulating the behaviour of proteins and starch subjected to mechanical stress and temperature changes during kneading and cooking (*36*). In a typical curve, the initial steps show the characteristics of gluten, and the last steps show starch properties (*37*). WWF showed higher water absorption than white flour, as found by Barros *et al*. (*38*). The arabinoxylans present in wheat bran have great capacity to bind water due to the presence of hydrophilic groups, responsible for the increased absorption of water in wholewheat flour (*36*). Water absorption was greater in the samples obtained with roller and cyclonic mills; however, they required lower developing time.

**Table 3:** Mixolab parameters of wholewheat flour

Variety	Mill type	*w*(WA)/%	C1/min	S/min	C2/(N∙m)	C3/(N∙m)	C4/(N∙m)	C5/(N∙m)
Klein Rayo	Cyclonic	68.80	(5.2±0.3)^a^	(3.54±0.03)^b^	(0.35±0.01)^a^	(1.40±0.01)^a^	(1.07±0.01)^a^	(2.0±0.2)^a^
	Hammer	64.70	(9.39±0.02)^c^	(1.2±0.7)^a^	(0.48±0.01)^b^	(1.92±0.01)^c^	(1.48±0.01)^c^	(2.59±0.04)^b^
	Roller	71.20	(7.7±0.4)^b^	(1.8±0.5)^a^	(0.46±0.03)^b^	(1.60±0.01)^b^	(1.22±0.04)^b^	(2.0±0.2)^a^
Fuste	Cyclonic	61.30	(4.32±0.05)^a^	(5.0±0.3)^b^	(0.44±0.01)^a^	(1.75±0.01)^a^	(1.41±0.00)^a^	(2.35±0.02)^b^
	Hammer	56.50	(10.4±0.1)^c^	(1.1±0.1)^a^	(0.54±0.01)^b^	(2.15±0.04)^b^	(1.80±0.01)^b^	(3.03±0.01)^b^
	Roller	64.85	(8.0±0.3)^b^	(1.6±0.2)^a^	(0.48±0.01)^a^	(1.73±0.01)^a^	(1.34±0.03)^a^	(2.30±0.05)^a^
INTA 815	Cyclonic	62.50	(3.0±0.3)^a^	(1.8±0.1)^a^	(0.37±0.00)^a^	(1.54±0.00)^a^	(1.05±0.04)^a^	(1.94±0.03)^a^
	Hammer	56.30	(6.7±0.9)^c^	(2.6±0.1)^a^	(0.51±0.00)^b^	(2.02±0.00)^b^	(1.7±0.7)^b^	(2.74±0.00)^b^
	Roller	65.80	(5.59±0.06)^b^	(1.7±0.4)^a^	(0.41±0.00)^a^	(1.57±0.02)^a^	(1.05±0.06)^a^	(1.8±0.2)^a^
Mean*	Cyclonic	(64.2±3.6)^b^	(4.2±1.0)^a^	(3.4±1.5)^b^	(0.4±0.1)^a^	(1.6±0.2)^a^	(1.2±0.2)^a^	(2.1±0.2)^b^
	Hammer	(59.2±43)^a^	(8.8±1.7)^b^	(1.6±0.8)^a^	(0.51±0.03)^c^	(2.0±0.1)^b^	(1.6±0.1)^b^	(2.8±0.2)^b^
	Roller	(67.3±3.1)^b^	(7.1±1.2)^b^	(1.7±0.2)^a^	(0.45±0.03)^b^	(1.6±0.1)^a^	(1.2±0.4)^a^	(2.0±0.2)^a^

WA=water absorption, C1=dough developing time, S=stability of dough, C2=protein weakening, C3=starch gelatinization, C4=stability of hot starch paste, C5=starch gelling. Different letters within a column for the same wheat variety indicate that values are significantly different at p<0.05. *Values of each milling treatment represent the average of the three varieties

On the other hand, the samples obtained in the hammer mill had the lowest water absorption capacity and the highest developing time. This result could be mainly due to the particle shape in these samples, which had medium-sized surface and were polygonal and coarse with particles of endosperm adhered. As a consequence, the surface was non-porous and with few internal surfaces (*30*). In addition, these samples had high starch gelatinization, stability of hot starch paste and starch gelling values (parameters related to starch pasting properties). Similarly, the characteristics of the particles generated during hammer milling hindered the hydration and gluten developed in the dough, thus water was available for starch gelatinization and the consequent retrogradation. According to Mixolab results, the particle size of WWF had no significant effect on water absorption, since the samples from roller and cyclonic mills had similar water absorption ability, while particle size was in opposing extremes. Similar results were reported by Zhang and Moore (*39*), who described that coarse wheat bran (609 mm) had higher water-holding capacity than fine bran (278 mm), but as wheat bran of different particle sizes were mixed into the flour, the bran particle size did not show any effect on water absorption. Taking into account that WWF obtained in cyclonic and roller mills had greater mass fraction of damaged starch than that in hammer mill, it could also play a more important role in determining water-holding properties than that played by bran particle size, as Niu *et al.* (*40*) observed when wheat was subjected to superfine grinding.

However, small particle size decreased developing time and increased protein weakening (low protein weakening) due to lower interference of bran in the development of gluten network and a larger contact surface between dough components; hence hydration rate of gluten protein was greater and consequently gluten developed quicker (*41*). However, the interaction between polypeptide chains was weaker in WWF. Wang *et al*. (*42*) reported an increased dough stability with reduction of flour particle size. In this work, although stability showed no clear trend, WWF obtained in cyclonic mill had greater stability than other types of flour, but only of Klein Rayo and Fuste varieties, since INTA 815 showed no significant differences among milling types. As opposed to white flour, where high dough development time, protein weakening and stability indicate strong gluten and good breadmaking quality, in wholewheat flour these parameters were affected by other factors such as particle size and shape and presence of fibre. The effect of variety was negligible. Klein Rayo variety had the highest water absorption ability and lowest protein weakening (stability). The developing time showed no clear effect since the milling process was probably more significant.

### Specific bread volume

**Fig. 2:**
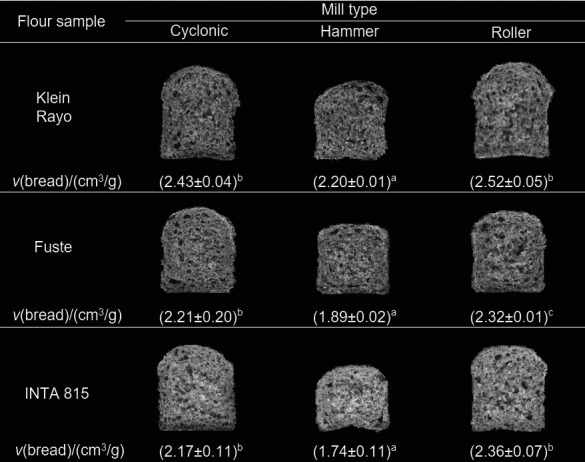
Representative images of microbread samples made with wholewheat flour obtained in different mills. Specific bread volumes (*V*) of the same variety followed by the same letter are not significantly different (p<0.05)

Fig. 2 shows microscale bread slices made with different WWF. The effect of milling type was significant; the WWF obtained in cyclonic and roller mills had higher specific volume than the flour made in the hammer mill. This bread showed a compact crumb insufficiently aerated with small cells. Conversely, roller and cyclonic mill bread had larger air cells and crumb was similar to that of white bread. The bread made with Klein Rayo flour had the highest specific volume regardless of the milling type, indicating that wheat with high protein content and breadmaking quality is needed to make wholemeal bread. In general, a comparison of these results with the literature is challenging since most of the studies were carried out with bran reincorporation, modified in particle size. The results reported in this work are opposite to Bressiani *et al.* (*4*), where they informed that WWF with medium particle size allowed higher specific bread volume than small and large particle size. However, these authors used an impact mill and different times of milling to obtain WWF of different particle size. The results of this work also differ from those of Noort *et al.* (*5*) since an increase in surface area by grinding did not lead to a decrease in specific bread volume.

Wang *et al.* (*42*) suggested that reducing particle size of WWF from ~160 to ~100 µm could be an effective way to improve the quality of whole wheat. In that work, the bran obtained with roller mill was further ground 1 to 4 times using a Perten laboratory mill. Thus, the used milling process was different from cyclonic milling.

The better performance of bread made with WWF in cyclonic and roller mills could be attributed to 30% higher water-soluble pentosan content in WWF samples than in those from the hammer mill. Water-soluble pentosans released during the breakdown of the kernel cell matrix probably played a key role in improving the bread quality by binding significant amounts of water. Thus, it resulted in less available water for starch gelatinization, allowing the loaves of bread to achieve higher volume before the breadcrumb structure was set.

### Dough thermogravimetric analysis

**Fig. 3:**
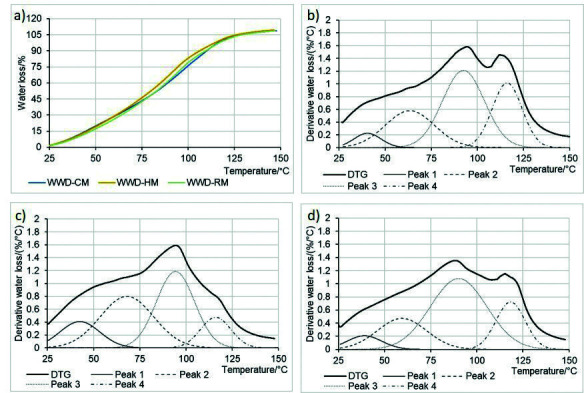
Thermogravimetric traces show: a) the effect of milling type on water loss (%) in wholewheat dough (WWD) of Klein Rayo variety from 25–150 °C and the first derivative thermogravimetry, representing the water loss rate (%/°C) of the WWD obtained by b) cyclonic, c) hammer and d) roller mills

Fig. 3a shows the thermograms of wholewheat dough water loss from Klein Rayo wholewheat flour obtained from different milling processes. As the samples had different optimal water absorption, all the thermogravimetric traces were normalized to the initial water content. When mass loss results from a single process, like dehydration, thermogravimetric traces show a sigmoid ascending trend with a flexus at some medium temperature where water loss rate is maximum (*43*). All samples exhibited a similar pattern. The flexus points were located around 92-95 °C where water loss of 72-74% took place.

The dough with wholewheat flour from hammer mill released a total of 75, 80 and 90% water at lower heating temperatures. The polygonal and coarse particles of wholewheat flour generated by this type of milling affected water distribution among the components in the dough samples and the amount of bound water decreased. This effect was also reflected at 90 °C, reaching a maximum temperature of bread crumb (*44*), where samples from hammer mill had higher percentages of water loss (65%) than the samples from cyclonic (58%) and roller mills (60%). An early decrease in water content could lead to premature settling of the crumb structure; therefore, it could limit the development of loaf volume.

Figs. 3b-3d show the first DTG plot obtained from the TGA data of each sample. The observed well-defined peaks correspond to the flexus points in thermogravimetric traces and suggest an increase in water evaporation rate (*45*). Maximum water loss rates ranged between 1.48 and 1.61%/°C. DTG profiles were influenced by the milling process. The dough with wholewheat flour from cyclonic and roller mills exhibited similar profiles. A mean peak around 95 °C and a secondary peak around 110 °C were observed. On the other hand, the secondary peak was absent from the dough with hammer-milled flour. Therefore, water loss rate at 110 °C was significantly lower in the dough samples with hammer-milled flour (Table 4). This may indirectly indicate in what manner the water is linked by dough components. Fessas and Schiraldi (*43*) studied the TGA profile of wheat dough and suggested that the presence of two peaks in the DTG profiles is attributed to different ways in which water is linked between the components of dough matrix. Free water was absorbed by gelatinized starch during temperature increase, and water strongly bound to the gluten network could only be evaporated at a higher temperature (>100 °C). Wholewheat flour is a heterogeneous system comprising polymers with different hydrophilic capacity (starch, non-starchy polysaccharides, fibre, gluten proteins, *etc*.), which therefore form separate aqueous phases, each with a particular composition (*46*). In our work, the deconvolution of each DTG profile of the dough samples allowed distinguishing an overview of water compartmentalization among matrix components (Figs. 3b-3d). The DTG profile of each sample was analysed with a 4-peak model. Adjusted model curves showed r^2^ values greater than 0.99. The first peak, whose maximum was around 42 °C, was attributed to the adsorbed water or weakly bound water to the bran particle surface, as suggested by Roozendaal *et al*. (*47*). The evaporation of this phase is linked to the low affinity of bran to water, which is released when placed under stress (*44*). The second peak, whose maximum was around 65 °C, refers to the water associated with starch, which is in agreement with both Fessas and Schiraldi (*43*) and Roozendaal *et al*. (*47*). Water is stored in the microcapillaries of starch granules and junction zones or held by hydrogen bonds between the amylose and amylopectin chains (*48*). Moreover, this water phase can be easily released when placed under mechanical stress or heating (*49*). A third peak, whose maximum was around 92 °C, was associated with water weakly bound to proteins, free to diffuse from the inside to the surface of the sample. Finally, a fourth peak above 110 °C was observed. According to Lapčíková e*t al.* (*50*), this fourth peak corresponds to the water strongly linked to gluten network. The magnitude of this stronger bond results from the resistance to the removal of this water from glutamine residues (*51*).

**Table 4:** Water loss (WL) content during wholewheat dough heating in different types of mill, water loss rate, maximum peak height temperature and associated area of each peak obtained by 4-peak deconvolution model of the DTG profile

Parameter		Cyclonic mill	Hammer mill	Roller mill
Temperature*/°C				
75% WL		(102.8±5.1)^b^	(96.0±0.3)^a^	(106.6±2.8)^b^
80% WL		(106.6±5.6)^b^	(99.4±0.4)^a^	(111.2±2.6)^b^
90% WL		(114.2±6.0)^b^	(108.0±0.5)^a^	(120.1±2.4)^b^
WL rate at 110 °C/(%/°C)		(1.37±0.02)^c^	(095±0.00)^a^	(1.04±0.03)^b^
1st peak	Max peak/°C	(41.9±1.1)^a^	(43.1±0.8)^a^	(41.3±3.1)^a^
	Area/%	(10.3±2.8)^a^	(12.3±2.0)^a^	(7.8±2.0)^a^
2nd peak	Max peak/°C	(64.4±1.3)^a^	(68.9±1.2)^a^	(62.1±3.7)^a^
	Area/%	(22.1±0.8)^a^	(36.7±1.1)^b^	(22.8±3.0)^a^
3rd peak	Max peak/°C	(92.3±0.2)^b^	(94.3±0.0)^c^	(90.9±1.1)^a^
	Area/%	(43.7±0.4)^a^	(38.3±0.2)^a^	(50.4±5.4)^b^
4th peak	Max peak/°C	(113.7±3.4)^a^	(116.4±0.4)^a^	(116.2±3.0)^a^
	Area/%	(23.9±3.2)^b^	(12.7±0.7)^a^	(19.1±0.5)^b^

*Temperature at which dough samples lose 75, 80 and 90% of water content. Values in the same row with common letter are not significantly different (p>0.05)

TGA test showed that the size and shape of flour particles obtained from different milling processes influenced water redistribution during baking. Table 4 shows average peak temperatures and relative peak area (in %). No significant differences in the maximum temperatures of each peak were observed. However, the milling type affected the peak area associated with water content bound to each component. The dough with the flour from hammer mill had a second peak with higher relative peak area. This milling type caused less particle damage and lower mass fraction of soluble pentosans; therefore, as there was more water available in the system, it increased the hydration of the starch granules during heating and the resulting gelatinization. These results are consistent with the starch gelatinization values obtained during Mixolab testing.

On the other hand, the flour from roller mill showed a third peak with a higher relative peak area. The particle morphology obtained by this mill type led to the formation of large insoluble protein aggregates and to an increase in the amount of water retained by this phase. In addition, cyclonic and roller mill dough had high relative area of the fourth peak, which could indicate more water bound to gluten and a well-developed network.

These findings suggest that particle size and shape of wholegrain flour obtained by different milling processes play a significant role in the water compartmentalization of the dough system. In addition, the magnitude of the processes involved in the baking, which are all governed mostly by water availability, may influence the final quality of the baked product. Nevertheless, both fine and large bran particle sizes seem to have the same effect on the water properties in wholewheat flour dough during heating.

## CONCLUSIONS

In this work, the study of wholewheat flour obtained through different mill types has allowed determining that wholegrain particle shape after milling has the main effect on product quality. In this sense, wheat grains milled in hammer mill had medium-size particles, but a portion of endosperm was adhered to the bran layers. As a consequence, these particles had lower content of damaged starch, wet gluten and soluble pentosans. In addition, these particle types increased hydration time, modified water distribution between flour components and hindered the appropriate development of dough; therefore, specific bread volume was low. On the other hand, in this work we demonstrated that particle size does not significantly influence the wholewheat flour quality. Both, the small particles obtained in cyclonic mill and the large particles obtained in roller mill had similar properties. These two milling types produce particles with thin layers of bran, completely separated from the endosperm, allowing a better water distribution between the dough components and improving gluten development, leading to higher specific bread volume. The effect of milling type and particle shape of the wholewheat flour had a greater effect than the wheat variety. Thus, the wholegrain milling process should be carefully selected taking to account the shape of the produced particle. Nevertheless, further research is needed to identify the main factors and particular components responsible for the detrimental effects on bread quality. This may open new opportunities for developing wholewheat bread with better acceptance by consumers.
